# Polygenic risk scores for Alzheimer's disease are related to dementia risk in APOE ɛ4 negatives

**DOI:** 10.1002/dad2.12142

**Published:** 2021-01-22

**Authors:** Jenna Najar, Sven J. van der Lee, Erik Joas, Hanna Wetterberg, John Hardy, Rita Guerreiro, Jose Bras, Margda Waern, Silke Kern, Henrik Zetterberg, Kaj Blennow, Ingmar Skoog, Anna Zettergren

**Affiliations:** ^1^ Department of Psychiatry and Neurochemistry Neuropsychiatric Epidemiology Unit Institute of Neuroscience and Physiology the Sahlgrenska Academy Centre for Ageing and Health (AGECAP) at the University of Gothenburg Mölndal Sweden; ^2^ Region Västra Götaland Sahlgrenska University Hospital, Psychiatry, Cognition and Old Age Psychiatry Clinic Gothenburg Sweden; ^3^ Section Genomics of Neurdegenerative Diseases and Aging Department of Clinical Genetics Vrije Universiteit Amsterdam, Amsterdam UMC Amsterdam The Netherlands; ^4^ Alzheimer Center Amsterdam Department of Neurology, Amsterdam Neuroscience Vrije Universiteit Amsterdam, Amsterdam UMC Amsterdam The Netherlands; ^5^ Department of Neurodegenerative Disease UCL Institute of Neurology London UK; ^6^ UK Dementia Research Institute at UCL London UK; ^7^ Reta Lila Weston Institute UCL Queen Square Institute of Neurology London UK; ^8^ NIHR University College London Hospitals Biomedical Research Centre London UK; ^9^ Institute of Advanced Study the Hong Kong University of Science and Technology Hong Kong SAR China; ^10^ Center for Neurodegenerative Science Van Andel Institute Grand Rapids Michigan USA; ^11^ Region Västra Götaland Sahlgrenska University Hospital, Psychosis Clinic Gothenburg Sweden; ^12^ Department of Psychiatry and Neurochemistry Institute of Neuroscience and Physiology The Sahlgrenska Academy at the University of Gothenburg Mölndal Sweden; ^13^ Clinical Neurochemistry Laboratory Sahlgrenska University Hospital Mölndal Sweden

**Keywords:** Alzheimer's disease, apolipoprotein E genotype, dementia, polygenic risk score, risk factors in epidemiology

## Abstract

**Introduction:**

Studies examining the effect of polygenic risk scores (PRS) for Alzheimer's disease (AD) and apolipoprotein E (*APOE*) genotype on incident dementia in very old individuals are lacking.

**Methods:**

A population‐based sample of 2052 individuals ages 70 to 111, from Sweden, was followed in relation to dementia. AD‐PRSs including 39, 57, 1333, and 13,942 single nucleotide polymorphisms (SNPs) were used.

**Results:**

AD‐PRSs (including 39 or 57 SNPs) were associated with dementia (57‐SNPs AD‐PRS: hazard ratio 1.09, confidence interval 1.01–1.19, *P *= .03), particularly in *APOE* ɛ4 non‐carriers (57‐SNPs AD‐PRS: 1.15, 1.05–1.27, *P = *4 × 10^–3^, 39‐SNPs AD‐PRS: 1.22, 1.10–1.35, *P = *2 × 10^–4^). No association was found with the other AD‐PRSs. Further, *APOE* ɛ4 was associated with increased risk of dementia (1.60, 1.35–1.92, *P *= 1 × 10^–7^). In those aged ≥95 years, the results were similar for the AD‐PRSs, while *APOE* ɛ4 only predicted dementia in the low‐risk tertile of AD‐PRSs.

**Discussion:**

These results provide information to identify individuals at increased risk of dementia.

## 1 BACKGROUND

In addition to the apolipoprotein E (*APOE*) gene, Alzheimer's disease (AD)‐associated single nucleotide polymorphisms (SNPs) have been identified through genome‐wide association studies (GWASs).^1^, ^2^, ^3^ These variants are usually combined into polygenic risk scores (PRSs), representing the accumulated effect of variants associated with AD.^4^ PRSs for AD (AD‐PRS) have been associated with AD and dementia in clinical^5^, ^6^, ^7^, ^8^, ^9^ and population‐based samples.^10^, ^11^, ^12^, ^13^ Thus far, population‐based studies mainly included individuals around age 60 years and the number of participants above age 95 years was relatively low.

Previous studies examining the effect of *APOE* genotype^14^, ^15^, ^16^ and other candidate genes^17^, ^18^ on cognitive impairment among the oldest old have generated conflicting results. The 90+ study (hazard ratio [HR] 1.04; 95% confidence interval [CI] 0.71–1.53) and the Vantaa 85+ study (HR 1.78; 95% CI 0.88–3.60) did not find an effect of the *APOE* ɛ4 allele on dementia risk,^14^, ^16^ while the Leiden 85+ study found an increased dementia risk among *APOE* ɛ4 carriers (odds ratio [OR] 4.1; 95% CI 2.1–8.4).^15^ The Rotterdam study reported an effect of AD‐PRS (including 23 SNPs) on dementia, with the strongest effects in *APOE* ɛ4 carriers.^10^ However, none of these studies investigated the effect of AD‐PRSs and the possible interaction with *APOE* genotypes on dementia risk in very old individuals.

We examined the effect of AD‐PRSs and *APOE* genotype on incident dementia, in a large population‐based study of individuals aged 70 to 111 years. We studied if the AD‐PRSs, the *APOE* genotype, and the interaction of these, predicted risk of dementia by analyzing the full age spectrum, as well as subgroups aged 70 to 94 years and 95 years or older.

## 2 METHODS

### 2.1 Study population

We used a population‐based sample of 3612 participants (1257 men, 2355 women), aged 70 to 111 years, with genotyped data from the Gothenburg H70 Birth Cohort Studies (including the H70, H75, H85, and H95+ studies, and the Prospective Population Study of Women). The participants were all residents of Gothenburg, Sweden, and were born 1901–1911, 1914, 1918, 1922–1924, 1930, or 1944. They were systematically selected from the Swedish Population Registry based on specific birth dates to yield representative samples at the ages studied.^19^, ^20^, ^21^, ^22^, ^23^ All participants were examined at least once between 2000 and 2016. Age at first examination ranged from 70 to 100 years.

**FIGURE 1:**
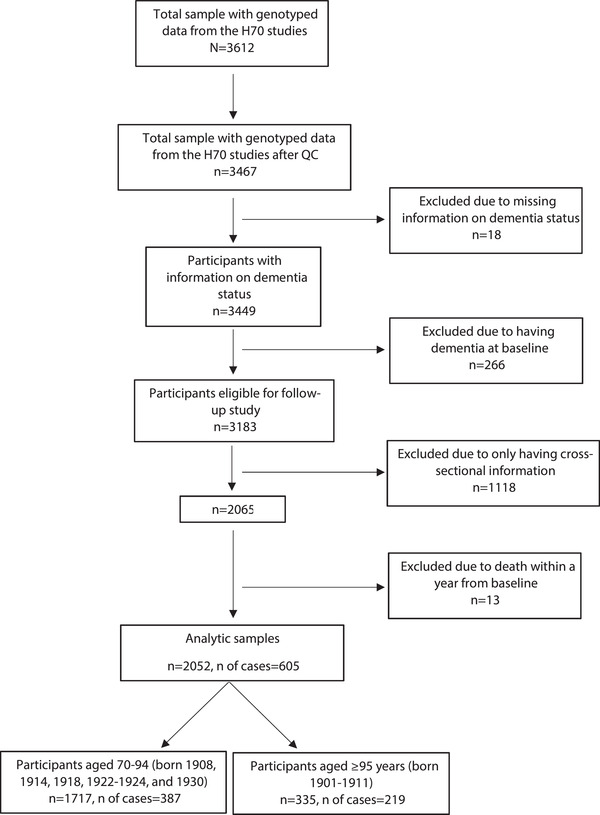
Flowchart of the participants included from the Gothenburg H70 Birth Cohort Study in the analytic samples of the present study (total analytic sample, and age subgroups)

In total, 3467 of 3612 had genotyped data after performing quality control (QC), of which 3449 (1196 men and 2253 women) had data on dementia status (see assessment procedures, sections 2.6 and 2.7). After exclusion of 266 persons with dementia at baseline, 3183 participants were eligible for the present follow‐up study. Of these, 1118 were excluded due to having cross‐sectional information only and 13 died within a year of baseline, leaving 2052 participants for the analytic sample (Figure 1).

The total sample was stratified based on age at blood sampling (70–94 years and ≥95 years). In those aged 70 to 94 years, 1717 were followed in relation to incident dementia and 387 developed dementia. Among those aged ≥95 years, 335 were followed in relation to incident dementia and 219 developed dementia (Figure 1).

### 2.2 Standard protocol approvals, registrations, and patient consents

The Ethics Committee for Medical Research at the University of Gothenburg approved the study and all participants gave informed consent to participate according to the Declaration of Helsinki.

**RESEARCH IN CONTEXT**

1. **Systematic review**: The authors used PubMed to identify previous studies examining polygenic risk scores (PRSs) in relation to Alzheimer's disease (AD) and dementia. However, population‐based studies examining the effect of AD‐PRSs and *APOE* genotype on incident dementia in samples of very old individuals are lacking.
2. **Interpretation**: In a population‐based sample of 2052 individuals aged 70 to 111 years, we found that AD‐PRSs (including 39 or 57 genetic variants) were associated with incident dementia, particularly in *APOE* ɛ4 non‐carriers and in those aged 95 years or older. *APOE* ɛ4 carriership was associated with increased, and ɛ2 carriership with reduced risk of dementia. Among those aged 95 years or older, *APOE* ɛ4 predicted dementia only in the low‐risk tertile of the AD‐PRSs.
3. **Future directions**: Results from this type of study could provide additional information to identify individuals at risk of dementia for implementation of potential preventative strategies before dementia pathology starts to accumulate.

### 2.3 Genetic analysis

Blood sampling for genetic analyses was performed in 2000–2011 and 2014–2016. Mean age at blood sampling was 80 years (standard deviation [SD] 9.5 years). Genotyping was performed with the NeuroChip (Illumina).^24^ QC included the removal of participants due to any of the following: per‐sample call rate <98%, sex mismatch, and excessive heterozygosity (FHET [F coefficient estimate for assessing heterozygosity] outside ± 0.2). Samples were defined as non‐European ancestral outliers, and removed, if their first two principal components (PCs) exceeded six standard deviations from the mean values of the European samples in the 1000 Genomes Project global reference population. Closely related samples were removed based on pairwise PI_HAT (i.e., proportion of genome that are in identity‐by‐descent; calculated using – genome option in PLINK) ≥0.2 (first‐ and second‐degree relatives). Further, markers were excluded due to per‐SNP call rate <98%, minor allele frequency (MAF) <0.01, and Hardy‐Weinberg disequilibrium (*P *< 1 × 10^‐6^). The Sanger imputation service was used to impute post‐QC, using the reference panel of Haplotype Reference Consortium data (HRC1.1). Post‐imputation QC included removal of SNPs with low imputation quality (RSQ [imputation quality] ≤0.3). The mean RSQ for the SNPs included in the AD‐PRSs was 0.83 (SD 0.13).

The variants rs7412 and rs429358 (which define the ε2, ε3, and ε4 alleles) in the *APOE* gene were also genotyped with the KASPar PCR SNP genotyping system (LGC Genomics, Hoddesdon, Herts, UK) or by mini‐sequencing, as previously described in detail.^25^

### 2.4 Polygenic risk scores

AD‐PRSs were generated using summary statistics from stage 1 of the most recent AD GWAS including clinically defined AD.^2^ SNPs were selected using linkage disequilibrium (LD) clumping. In short, the European ancestry samples from the 1000 Genomes Project were used as a reference panel to remove variants in LD, all variants 250 kb upstream and downstream of top signal were removed (R^2 ^<0.001). All variants in the *APOE* region (chromosome 19, coordinates hg19 [GRCh37]: 44412079 to 46412079) were removed. In the present study, we created PRSs including variants that surpassed four *P* value thresholds (*P <* 1e^–5^, *P <* 1e^–3^, *P <* 1e^–1^), referred to as 1e^–5^ AD‐PRS (including 57 SNPs, Table S1 in supporting information), 1e^–3^ AD‐PRS (including 1333 SNPs), and 1e^–1^ AD‐PRS (including 13,942 SNPs). For the *P* < 5e^–8^ level we used an AD‐PRS based on 39 SNPs (39‐SNPs AD‐PRS, Table S1) that have shown genome‐wide significant association with AD after combined meta‐analyses in the most recent GWAS by de Rojas et al.^3^ In the same study, the 39‐SNPs AD‐PRS was also validated for the first time in a clinical sample.^3^ All AD‐PRSs were calculated as the sum of the β‐coefficient multiplied with the number (or dosage) of effect alleles of each SNP.

The population was further divided into low‐, middle‐, and high‐risk tertiles of AD‐PRSs. To avoid boundaries being affected by survival to old age, limits of the tertiles were calculated using data from 1130 participants born 1944 (mean age at blood sampling 70.6 years, SD 0.3 years). The AD‐PRSs were standardized and used as continuous variables in all analyses, while tertiles of AD‐PRSs were used to stratify the data.

### 2.5 APOE genotype

*APOE* genotype was divided into ɛ4 carriers (ɛ4*/*ɛ2, ɛ4*/*ɛ3, or ɛ4*/*ɛ4) and ɛ4 non‐carriers (ɛ2/ɛ2, ɛ3/ɛ3, or ɛ3/ɛ2), as well as into ɛ2 carriers (ɛ2/ɛ2, ɛ2/ɛ3, ɛ2/ɛ4) and ɛ2 non‐carriers (ɛ3/ɛ3, ɛ3/ɛ4, ɛ4/ɛ4).

In sensitivity analyses, we excluded ɛ4/ɛ2 heterozygotes. Thus, *APOE* genotype was divided into ɛ4 carriers comprising ɛ4*/*ɛ3 heterozygotes and ɛ4*/*ɛ4 homozygotes, and ɛ2 carriers comprising ɛ3/ɛ2 heterozygotes and ɛ2/ɛ2 homozygotes.

### 2.6 Neuropsychiatric examination

Neuropsychiatric examinations were performed by experienced psychiatric nurses in 2000–2016. The examinations were semi‐structured and included comprehensive neuropsychiatric examinations and an extensive battery of neuropsychological tests.^26^ Close informant interviews were performed by psychiatric nurses or psychologists. The interviews were semi‐structured and comprised questions about changes in behavior and intellectual function; psychiatric symptoms; activities of daily living; and in cases of dementia, age of onset and disease course.^27^

### 2.7 Diagnosis of dementia

The diagnosis of dementia at each examination was based on Diagnostic and Statistical Manual of Mental Disorders Third Edition‐Revised (DSM‐III‐R) criteria,^28^ using information from neuropsychiatric examinations and close informant interviews.^26^, ^27^ Dementia diagnoses for individuals lost to follow‐up were based on information obtained from the Swedish Inpatient Registry until 2012.^26^ In the present study, 441 (72.9%) of 605 dementia diagnoses were based on neuropsychiatric examinations.

Age of dementia onset was based on information provided by close informants, the examinations, and the Swedish Inpatient Register. If no information could be obtained from these sources, the age of onset was determined as the mid‐point between the last examination at which dementia criteria were not fulfilled and the first with a dementia diagnosis. Information on deaths during follow‐up was obtained from the Swedish Population Registry until December 31, 2016.

### 2.8 Statistical analysis

All analyses were done in R (version 4.0.0) using the survival and survminer packages. Sample characteristics are presented as numbers, mean and median values, SD, minimum (min) and maximum (max) values, and percentages. Information on years of education and Mini‐Mental State Examination (MMSE) score was obtained through semi‐structured interviews at age of blood sampling.

Cox regression models using age as time scale were used to analyze the effect of the AD‐PRSs and *APOE* genotype on incident dementia, presented as HR and 95% CI in a model including the following covariates: age at blood sampling, birth year, sex, and 10 PCs to correct for population stratification. Participants were censored at the date of (1) dementia diagnosis, (2) death, or (3) end of follow‐up (December 31, 2016 for those with last examination year in 2015–2016, and December 31, 2012 for those with last examination year in 2009–2010, and register data until 2012). The proportional hazard assumption was verified using Schoenfeld residuals.

We examined the interaction of *APOE* genotype (based on ɛ4 or ɛ2 carriership in separate models) and AD‐PRSs in relation to incident dementia. Further, based on the results of the interaction analyses, we examined the effect of the AD‐PRSs stratified by *APOE* ɛ4 carriership, and the effect of *APOE* ɛ4 carriership stratified by tertiles of 39‐SNPs AD‐PRS and 1e^–5^ AD‐PRS, respectively. The analyses were carried out in the total sample, and in those aged 70–94 years and ≥95 years.

In a subsample of individuals with genotyped data and information on MMSE and years of education (n = 1394), we repeated the analyses of *APOE* genotype and the 39‐SNPs AD‐PRS, and 1e^–5^ AD‐PRS in relation to incident dementia using Cox regression models adjusted for age at blood sampling, birth year, sex, 10 PCs, MMSE score, and years of education.

Furthermore, we examined the effect of the 39‐SNPs AD‐PRS, the 1e^–5^ AD‐PRS, and *APOE* genotype on mortality in a Cox regression model adjusted for age at blood sampling, birth year, sex, and 10 PCs. We also examined the interaction between *APOE* ɛ4 status and the two AD‐PRSs in relation to mortality.

Finally, we examined the individual effect of the different SNPs included in the 39‐SNPs AD‐PRS and 1e^–5^ AD‐PRS on incident dementia in Cox regression models using age as time scale, adjusted for age at blood sampling, birth year, sex, and 10 PCs. Further, to examine whether any of those SNPs drove the associations with the AD‐PRSs, analyses were performed with the exclusion of the SNPs associated with incident dementia, one by one.

## 3 RESULTS

Sample characteristics by *APOE* genotype and tertiles of the 39‐SNPs AD‐PRS and the 1e^–5^ AD‐PRS are shown in Table 1. During a mean follow‐up of 7.2 years (SD 4.7 years; 14,775 person‐years), 605 participants developed dementia, with mean age of dementia onset 89.4 years (SD 8.2 years). In total, 1243 participants were censored due to death (72.5% women) with a median age at death of 90 years (range, 71–111 years). Compared to those excluded (n = 1131, Figure 1), participants who were followed up (n = 2052) had a higher mean age at baseline (*P *< .001), were more likely women (*P *< .001), and had a lower frequency of *APOE* ɛ4 carriers (*P *< .001), while no differences were found in AD‐PRSs (the 39‐SNPs AD‐PRS and 1e^–5^ AD‐PRS) or median age at death. Compared to those with prevalent dementia (n = 266, Figure 1), individuals with incident dementia (n = 605) had a higher age at death (*P *< .001) and a lower frequency of *APOE* ɛ4 carriers (*P *= .049), while no differences were found in sex, the AD‐PRSs (the 39‐SNPs AD‐PRS and 1e^–5^ AD‐PRS), or age at baseline.

**TABLE 1:** Sample characteristics by APOE ɛ4 allele and tertile of AD‐PRSs

		*APOE* genotype		39‐SNPs AD‐PRS tertile			1e^–5^ tertile AD‐PRS		
Characteristics	Total sample	*APOE* ɛ4 carriers	*APOE* ɛ4 non‐carriers	Low‐risk	Middle‐risk	High‐risk	Low‐risk	Middle‐risk	High‐risk
	(n = 2052)	(n = 505)	(n = 1547)	(n = 652)	(n = 653)	(n = 747)	(n = 723)	(n = 638)	(n = 691)
Age at blood sampling (years), mean (SD)	80 (9.5)	78.7 (8.8)	80.6 (9.7)	80.3 (9.9)	80.6 (9.4)	79.5 (9.2)	80.5 (9.8)	79.7 (9.3)	80.1 (9.3)
Education (years), mean (SD)^a^	9.7 (5.4)	10.1 (6.2)	9.5 (5.1)	10.0 (5.0)	9.6 (6.1)	9.5 (5.2)	9.9 (5.2)	9.8 (6.1)	9.3 (4.9)
*APOE* ɛ4 *c*arriership,% (cases/total)	24.6 (505/2052)	NA	NA	48.9 (319/652)	18.5 (121/653)	8.7 (65/747)	25.4 (184/723)	22.4 (143/638)	25.8 (178/691)
*APOE* ɛ*2 c*arriership,% (cases/total)	14.8 (303/2052)	7.3 (37/505)	17.2 (266/1547)	12/1 (79/652)	14.4 (94/653)	17.4 (130/747)	14.0 (101/753)	16.5 (105/638)	14.0 (97/691)
39‐SNPs AD‐PRS, median (min, max)	0.1 (–3.5, 4.1)	–0.8 (–3.5, 1.6)	0.3 (–3.1, 4.1)	–1.0 (–3.5, –0.5)	0.003 (–0.5, 0.4)	0.9 (0.4, 4.1)	–0.5 (–3.5, 2.3)	0.1 (–2.9, 3.1)	0.6 (–1.9, 4.1)
1e^–5^ AD‐PRS, median (min, max)	–0.1 (–3.6, 3.7)	–0.1 (–2.9, 3.7)	–0.1 (–3.6, 3.4)	–0.6 (–3.6, 3.3)	–0.1 (–2.5, 3.7)	0.5 (–2.8, 3.4)	–1.0 (–3.6, –0.4)	–0.1 (–0.4, 0.4)	0.9 (0.4, 3.7)
Sex female, % (cases/total)	70.3 (1442/2052)	70.5 (356/505)	70.2 (1086/1547)	70.2 (458/652)	73.0 (477/653)	67.9 (507/747)	70.7 (511/723)	69.4 (443/638)	70.5 (487/691)
Dementia diagnosis, % (cases/total)	29.5 (605/2052)	36.0 (182/505)	27.3 (423/1547)	31.0 (202/652)	27.7 (181/653)	29.7 (222/747)	28.8 (208/723)	28.2 (180/638)	31.4 (217/691)
Age at dementia onset (years), mean (SD)	89.4 (8.2)	87.3 (7.8)	90.3 (8.2)	89.7 (8.3)	89.4 (7.8)	89.1 (8.4)	90.2 (8.0)	88.6 (8.5)	89.2 (8.1)
MMSE score at baseline, median (min, max)^b^	28 (0, 30)	28 (2, 30)	28 (0, 30)	28 (2, 30)	28 (0, 30)	28 (0, 30)	28 (0, 30)	28 (0, 30)	28 (0, 30)
Mortality during follow‐up, % (cases/total)	60.6 (1243/2052)	61.4 (310/505)	60.3 (933/1547)	63.0 (411/652)	60.3 (394/653)	58.6 (438/747)	63.9 (462/723)	56.3 (359/638)	61.1 (422/691)
Age at death (years), median (min, max)	90.0 (71, 111)	89.0 (72, 106)	91.0 (71, 111)	90.0 (72, 111)	90.0 (71, 107)	90.0 (72, 107)	91 (72, 111)	90 (73, 107)	90 (71, 106)

^a^ n = 1471, information on years of education was obtained through semi‐structured interviews at age of blood sampling.

^b^ n = 1669, MMSE score was obtained through neuropsychiatric examinations at age of blood sampling.

Abbreviations: AD, Alzheimer's disease; APOE, apolipoprotein E; MMSE, Mini‐Mental State Examination; PRS, polygenic risk score; SD, standard deviation; SNP, single nucleotide polymorphism.

**FIGURE 2:**
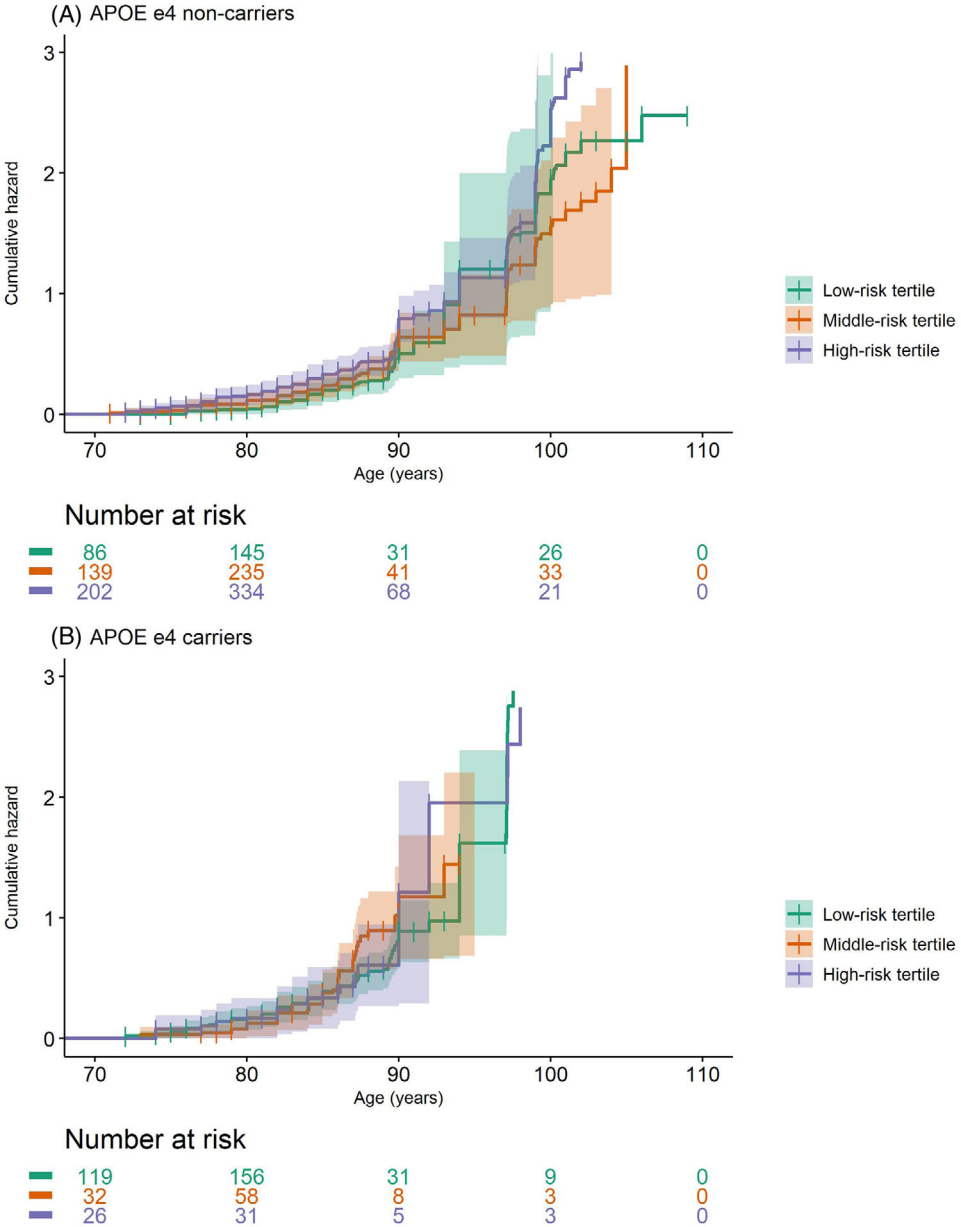
Cumulative hazard of dementia by tertile of the 39‐single nucleotide polymorphisms (SNPs) Alzheimer's disease (AD)‐polygenic risk scores (PRS; low‐risk tertile, middle‐risk tertile, and high‐risk‐tertile) stratified by apolipoprotein E (*APOE*) ɛ4 carriership (A, *APOE* ɛ4 non‐carriers, and B, *APOE* ɛ4 carriers). Analysis adjusted for covariates (age at blood sampling, birth year, sex, and 10 principal components to correct for population stratification) set to sample average

**FIGURE 3:**
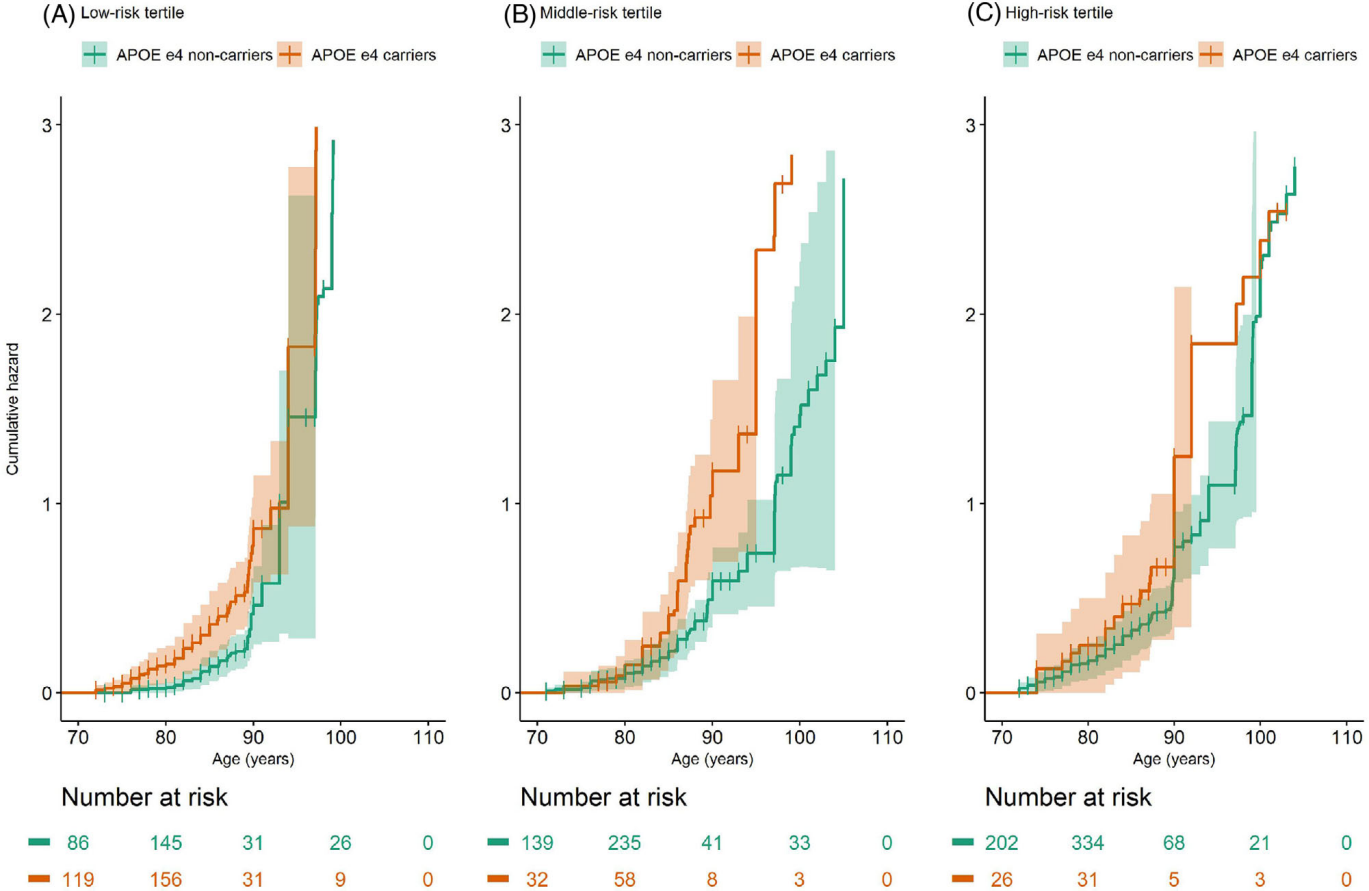
Cumulative hazard of dementia by *APOE* ɛ4 carriership (*APOE* ɛ4 carriers and non‐carriers), stratified by tertile of the 39‐single nucleotide polymorphisms (SNPs) Alzheimer's disease (AD)‐polygenic risk scores (PRS; A, low‐risk tertile, B, middle‐risk tertile, and C, high‐risk tertile). Analysis adjusted for covariates (age at blood sampling, birth year, sex, and 10 principal components to correct for population stratification) set to sample average

In all individuals with follow‐up data (n = 2052), *APOE* ɛ4 carriership (see Table 3; HR 1.60; 95% CI 1.35–1.92, *P *= 1 × 10^–7^) and the 1e^–5^ AD‐PRS (see Table 2; HR 1.09; 95% CI 1.01–1.19, *P *= .03) were associated with increased risk of dementia, while ɛ2 carriership was associated with reduced risk (HR 0.74; 95% CI 0.59–0.95, *P *= .02). No association was found between the 39‐SNPs AD‐PRS (see Table 2; HR 1.03; 95% CI 0.95–1.11, *P = *.5), 1e^–3^ AD‐PRS (HR 1.05; 95% CI 0.97–1.14, *P = *.2), and 1e^–1^ AD‐PRS (HR 0.99; 95% CI 0.91–1.08, *P = *.8) and incident dementia. However, there was an interaction between *APOE* ɛ4 carriership and the 39‐SNPs AD‐PRS (*P *= .02), and between *APOE* ɛ4 status and the 1e^–5^ AD‐PRS (*P *= .05) in relation to incident dementia. No interaction was observed between *APOE* ɛ4 carriership and the 1e^–3^ AD‐PRS (*P *= .6) or the 1e^–1^ AD‐PRS (*P *= .4) in relation to incident dementia. Stratified analysis showed that the 39‐SNPs AD‐PRS and the 1e^–5^ AD‐PRS were associated with dementia among *APOE* ɛ4 non‐carriers (39‐SNPs AD‐PRS: HR 1.22; 95% CI 1.10–1.35, *P *= 2 × 10^–4^_,_ 1e^–5^ AD‐PRS: HR 1.15, 95% CI 1.05–1.27, *P *= 4 × 10^–3^), while no association was found among ɛ4 carriers (Table 2, Figure 2). After stratifying by the AD‐PRSs tertiles, *APOE* ɛ4 carriership was only associated with incident dementia among those in the low‐ and middle‐risk tertiles (Table 3, Figure 3). No interaction was found between *APOE* ɛ2 carriership and the 39‐SNPs AD‐PRS (*P *= .6) or the 1e^–5^ AD‐PRS (*P* = .3). In sensitivity analyses, *APOE* ɛ4 (HR 1.68; 95% CI 1.41–2.02, *P = *1 × 10^–8^) and ɛ2 carriership (HR 0.76; 95% CI 0.59–0.97, *P = *.03) remained associated with incident dementia, after excluding *APOE* ɛ4/ɛ2 heterozygotes (n = 2015).

**TABLE 2:** Associations between AD‐PRSs (the 39‐SNPs AD‐PRS and the 1e^–5^ AD‐PRS) and incident dementia, stratified by age groups and *APOE* ɛ4 carriership

	Total sample (n = 2052)			
	HR^a^	CI	*P* value	Number of events
39‐SNPs AD‐PRS	1.03	0.95‐1.11	.5	605
39‐SNPs AD‐PRS in *APOE* ɛ4 non‐carriers	1.22	1.10‐1.35	2 × 10^–4^	423
39‐SNPs AD‐PRS in *APOE* ɛ4 carriers	0.94	0.79‐1.12	.5	182
				
1e^–5^ AD‐PRS	1.09	1.01‐1.19	.03	605
1e^–5^ AD‐PRS in *APOE* ɛ4 non‐carriers	1.15	1.05‐1.27	4 × 10^–3^	423
1e^–5^ AD‐PRS in *APOE* ɛ4 carriers	0.94	0.81‐1.09	.4	182
	**70‐94 years (n = 1717)**			
39‐SNPs AD‐PRS	0.99	0.89–1.09	.8	386
39‐SNPs AD‐PRS in *APOE* ɛ4 non‐carriers	1.16	1.01–1.34	.03	247
39‐SNPs AD‐PRS in *APOE* ɛ4 carriers	1.08	0.88–1.33	.5	139
				
1e^–5^ AD‐PRS	1.07	0.96–1.19	.2	386
1e^–5^ AD‐PRS in *APOE* ɛ4 non‐carriers	1.18	0.98–1.27	.1	247
1e^–5^ AD‐PRS in *APOE* ɛ4 carriers	0.99	0.83–1.17	.9	139
	**95+ years (n = 335)**			
39‐SNPs AD‐PRS	1.12	0.98–1.29	.1	219
39‐SNPs AD‐PRS in *APOE* ɛ4 non‐carriers	1.28	1.10–1.50	2 × 10^–3^	176
39‐SNPs AD‐PRS in *APOE* ɛ4 carriers	0.62	0.41–0.95	.03	43
				
1e^–5^ AD‐PRS	1.15	1.01–1.32	.04	219
1e^–5^ AD‐PRS in *APOE* ɛ4 non‐carriers	1.12	0.98–1.27	.1	176
1e^–5^ AD‐PRS in *APOE* ɛ4 carriers	0.75	0.52–1.08	.1	43

Notes: Stage 1 of the most recent AD GWAS (Kunkle et al.^2^) was used to generate the AD‐PRSs.

^a^ HR per standard deviation (SD) of the score.

Abbreviations: AD, Alzheimer's disease; APOE, apolipoprotein E; GWAS, genome‐wide association study; HR, hazard ratio; PRS, polygenic risk score; SNP, single nucleotide polymorphism.

**TABLE 3:** Associations between *APOE* ɛ4 allele and incident dementia, stratified by age groups and tertiles of AD‐PRSs

	Total sample (n = 2052)			
	HR	CI	*P* value	Number of events
*APOE* ɛ4	1.60	1.35–1.92	1 × 10^–7^	605
*APOE* ɛ4 in low‐risk 39‐SNPs AD‐PRS	1.98	1.47–2.66	7 × 10^–6^	202
*APOE* ɛ4 in middle‐risk 39‐SNPs AD‐PRS	2.00	1.43–2.81	6 × 10^–5^	181
*APOE* ɛ4 in high‐risk 39‐SNPs AD‐PRS	1.18	0.75–1.84	.48	222
				
*APOE* ɛ4 in low‐risk 1e^–5^ AD‐PRS	2.20	1.62–3.00	5 × 10^–7^	208
*APOE* ɛ4 in middle‐risk 1e^–5^ AD‐PRS	1.41	1.01–1.96	.04	180
*APOE* ɛ4 in high‐risk 1e^–5^ AD‐PRS	1.35	0.99–1.82	.05	217
	**70‐94 years (n = 1717)**			
*APOE* ɛ4	1.75	1.42–2.16	2 × 10^–7^	386
*APOE* ɛ4 in low‐risk 39‐SNPs AD‐PRS	2.01	1.37–2.95	4 × 10^–4^	124
*APOE* ɛ4 in middle‐risk 39‐SNPs AD‐PRS	2.39	1.61–3.55	2 × 10^–5^	117
*APOE* ɛ4 in high‐risk 39‐SNPs AD‐PRS	1.65	0.99–2.77	.06	145
				
*APOE* ɛ4 in low‐risk 1e^–5^ AD‐PRS	2.13	1.48–3.08	6 × 10^–5^	125
*APOE* ɛ4 in middle‐risk 1e^–5^ AD‐PRS	1.87	1.26–2.77	2 × 10^–3^	119
*APOE* ɛ4 in high‐risk 1e^–5^ AD‐PRS	1.44	1.01–2.06	.04	142
	**95+ years (n = 335)**			
*APOE* ɛ4	1.33	0.94–1.88	.11	219
*APOE* ɛ4 in low‐risk 39‐SNPs AD‐PRS	1.72	1.01–2.92	.05	78
*APOE* ɛ4 in middle‐risk 39‐SNPs AD‐PRS	1.27	0.55–2.95	.58	64
*APOE* ɛ4 in high‐risk 39‐SNPs AD‐PRS	0.79	0.30–2.09	.63	77
				
*APOE* ɛ4 in low‐risk 1e^–5^ AD‐PRS	3.66	1.99–6.73	3 × 10^–5^	83
*APOE* ɛ4 in middle‐risk 1e^–5^ AD‐PRS	0.71	0.35–1.43	.3	61
*APOE* ɛ4 in high‐risk 1e^–5^ AD‐PRS	1.06	0.55–2.02	.9	75

Note: Stage 1 of the most recent AD GWAS (Kunkle et al.^2^) was used to generate the AD‐PRSs.

Abbreviations: AD, Alzheimer's disease; APOE, apolipoprotein E; CI, confidence interval; HR, hazard ratio; PRS, polygenic risk score; SNP, single nucleotide polymorphism.

In a subsample of individuals with genotyped data and information on years of education and MMSE score (n = 1394), the main results described above did not change after including these variables as covariates (data not shown).

Among those aged 70 to 94 years, the 39‐SNPs AD‐PRS was associated with incident dementia in *APOE* ɛ4 non‐carriers (HR 1.16; 95% CI 1.01–1.34, *P *= .03; Table 2), but not in ɛ4 carriers (HR 1.08; 95% CI 0.88–1.33, *P *= .5; Table 2). The 1e^–5^ AD‐PRS was not associated with incident dementia in this age group (HR 1.07; 95% CI 0.96–1.19, *P *= .2; Table 2). *APOE* ɛ4 carriership predicted dementia in the low‐ and middle‐risk tertiles of the 39‐SNPs AD‐PRS, and in all tertiles of the 1e^–5^ AD‐PRS (Table 3). Moreover, *APOE* ɛ2 carriership was associated with reduced risk of dementia (HR 0.69; 95% CI 0.51–0.95, *P *= .02).

In those aged ≥95 years, 1e^–5^ AD‐PRS was associated with incident dementia (HR 1.15, 95% CI 1.01–1.32, *P *= .04), while the 39‐SNPs AD‐PRS was associated with incident dementia among *APOE* ɛ4 non‐carriers only (HR 1.28; 95% CI 1.10–1.50, *P *= 2 × 10^–3^; Table 2). In *APOE* ɛ4 carriers the 39‐SNPs AD‐PRS was instead related to a reduced risk of dementia (HR 0.62; 95% CI 0.41–0.95, *P *= .03; Table 2). *APOE* ɛ4 carriership only predicted dementia in the low‐risk tertile of the AD‐PRSs (39‐SNPs AD‐PRS: HR 1.72; 95% CI 1.01–2.92, *P *= .05, 1e^–5^ AD‐PRS: HR 3.66; 95% CI 1.99–6.73, *P *= 3 × 10^–5^; Table 3). No association was found between *APOE* ɛ2 carriership and dementia (HR 0.80; 95% CI 0.55–1.17, *P *= .3) in this age group.

Furthermore, *APOE* ɛ4 carriership was associated with increased risk of mortality (HR 1.21; 95% CI 1.07–1.39, *P *= 3 × 10^–3^), while ɛ2 carriership (HR 1.01; 95% CI 0.87–1.18, *P *= .8), the 39‐SNPs AD‐PRS (HR 1.0; 95% CI 0.87–1.15, *P *> .9), and the 1e^–5^ AD‐PRS (HR 1.03; 95% CI 0.97–1.09, *P = *.4) were not. There was no interaction between *APOE* ɛ4 allele and the 39‐SNPs AD‐PRS (*P *= .1) or the 1e^–5^ AD‐PRS (*P >* .9) in relation to risk of mortality.

The SNPs rs876461 (*PRKD3*; *P *= .02), rs117618017 (*APH1B*; *P *= .03), rs4844610 (*CR1*; *P *= .04), rs7584040 (*BIN1*; *P *= 2 × 10^–3^), rs11168036 (*HBEGF*; *P *= 8 × 10^–3^), rs143429938 (*CLU/MIR6843*; *P *= .04), and rs8064326 (*KCTD2*; *P *= 3 × 10^‐4^) were independently associated with incident dementia (Table S1). Among *APOE* ɛ4 non‐carriers, the 39‐SNPs AD‐PRS remained associated with incident dementia after removal of rs876461, rs117618017, and rs4844610 (for all analyses: *P *= 2 × 10^–4^). The 1e^–5^ AD‐PRS remained associated with incident dementia among *APOE* ɛ4 non‐carriers after removal of rs7584040 (*P *= .01), rs11168036 (*P *= 9 × 10^–3^), rs143429938 (*P *= 6 × 10^–3^), and rs8064326 (*P *= .01). However, the association between the 1e^–5^ AD‐PRS and incident dementia in the total sample (not stratified by *APOE* ɛ4 status) was slightly attenuated after removal of the SNPs (for all analyses: *P *= .06). Overall, our analyses indicated that the associations seen with the AD‐PRSs were not mainly driven by one, or a few, SNPs.

## 4 DISCUSSION

In a population‐based sample of individuals aged 70 to 111 years, AD‐PRSs (including 39 or 57 SNPs) were associated with incident dementia, particularly in *APOE* ɛ4 non‐carriers and in those aged 95 years or older. However, no association was found between the wider AD‐PRSs (including >1000 SNPs, *P* values ≥ 1e^–3^) and incident dementia. *APOE* ɛ4 carriership was associated with increased risk of dementia, especially in the low‐ and middle‐risk tertiles of the AD‐PRSs, while ɛ2 carriership was associated with reduced risk. Among those aged 95 years or older, *APOE* ɛ4 carriership only predicted dementia in the low‐risk tertile of the AD‐PRSs. To our knowledge, this is the first study reporting an effect of AD‐related genetic variants beyond that of *APOE* genotype on dementia risk in the oldest old.

Previous studies have reported an association between AD‐PRS and dementia in clinical^3^, ^5^, ^7^, ^9^ and population‐based samples.^10^, ^13^ However, the modifying effect of *APOE* ɛ4 status on the association between AD‐PRS and dementia varies; clinical samples report a similar effect of AD‐PRS on AD in *APOE* ɛ4 carriers and non‐carriers,^3^, ^5^, ^7^ while the population‐based Rotterdam Study reported a higher effect of an AD‐PRS on AD and dementia among *APOE* ɛ4 carriers compared to non‐carriers.^10^ We found an association between AD‐PRSs (including 39 and 57 SNPs) and dementia among *APOE* ɛ4 non‐carriers. One reason for the discrepancy could be that our study had a rather high mean age at baseline (80 years), whereas the Rotterdam Study had a considerably lower age at inclusion (mean 67.5 years).^10^ *APOE* ɛ4 carriership is related to earlier age of dementia onset^29^ as well as premature death.^30^ In the current study, the inclusion of older participants renders a selection of healthier *APOE* ɛ4 carriers who may carry additional genetic variants preventing them from developing dementia at the ages observed. Further, our AD‐PRSs included 39 SNPs and 57 SNPs, while the population‐based Rotterdam Study, reporting a higher effect among *APOE* ɛ4 carriers, used an AD‐PRS including 23 SNPs.^10^ However, analysis using this 23 SNPs AD‐PRS in our sample showed similar results as for the 39 SNPs AD‐PRS (data not shown).

We found no effect of the wider AD‐PRSs (including more than 1000 SNPs, *P* values ≥1e^–3^) on incident dementia. In contrast, Escott‐Price et al. reported that AD‐PRSs including genetic variants at *P* value ≤0.5 had the highest prediction of AD.^9^ Discrepancies could be due to differences in study setting and design, such as diagnostic status (i.e., dementia in our study and AD in the study by Escott‐Price et al.) and age at inclusion. Further, discrepancies could also arise due to differences in the construction of the PRSs (e.g., we used a rather strict R^2^ threshold [R^2 ^= 0.001] for clumping, while Escott‐Price et al. chose a more liberal level [R^2 ^= 0.2]).^9^

We did not find an effect of *APOE* ɛ4 carriership on dementia risk in participants aged 95 years or older. However, we found that the *APOE* ɛ4 allele predicted dementia in the low‐risk AD‐PRSs tertile. This finding indicates, once again, that very old dementia‐free survivors, carrying genetic high‐risk variants (i.e., both *APOE* ɛ4 and a high or medium risk based on the AD‐PRSs), probably carry undiscovered protective genetic variants. In line with this, we found that the AD‐PRS was associated with reduced risk of dementia in *APOE* ɛ4 carriers, while it was associated with increased risk of dementia in *APOE* ɛ4 non‐carriers. Consistent with other studies,^14^, ^31^, ^32^ we found no effect of *APOE* ɛ2 carriership on dementia risk in those aged 95 years or older.

Strengths of this study include the population‐based sample and a large subsample of participants aged 95 years and above. In addition, psychiatric nurses performed the neuropsychiatric examinations and multiple sources of information were used to detect and diagnose dementia according to established diagnostic criteria throughout the study. There are also some possible limitations. First, we cannot exclude that our results could have been affected by selection bias. Although the larger proportion of individuals aged 95 years or older can be considered a strength, our results should be interpreted with regard to the selection of healthier individuals at these higher ages. Second, there might be unidentified dementia cases in the control group. However, our frequent follow‐ups with neuropsychiatric examinations, standardized diagnostic procedures, and use of registers to detect dementia reduces this possibility. Third, cumulative attrition is a problem in long‐term prospective studies. Although this issue was partially alleviated by using the Swedish Inpatient Registry to detect dementia in those lost to follow‐up, these sources underestimate the number of dementia cases.^33^ However, it should be noted that almost all people in Sweden receive hospital treatment within the public health‐care system and that the Swedish Inpatient Registry covers the entire country. If anything, underestimation of dementia cases would reduce the possibility of finding true associations. Fourth, the AD‐PRSs comprise SNPs associated with AD. We examined the effect of the AD‐PRSs on incident dementia, which could include dementia subtypes other than AD, not as strongly linked to the SNPs included in the AD‐PRSs. This would most likely attenuate the associations. It is noteworthy that in Swedish populations, approximately two thirds of those with dementia have AD.^34^, ^35^ Fifth, *APOE* ɛ4 allele was associated with mortality and competing risk of death is likely to have affected the results. Sixth, all members of the sample are White and living in Sweden, thereby limiting the possibility to generalize to other populations.

In conclusion, we found an effect of AD‐PRSs (including 39 SNPs or 57 SNPs) and *APOE* genotype on dementia risk in the general population up to very old ages, especially among *APOE* ɛ4 non‐carriers. Results from this type of study could provide additional information to identify individuals at increased risk of dementia for implementation of potential preventative strategies before dementia pathology starts to accumulate.

## CONFLICTS OF INTEREST

Henrik Zetterberg has served at scientific advisory boards for Denali, Roche Diagnostics, Wave, Samumed, Siemens Healthineers, Pinteon Therapeutics, and CogRx; has given lectures in symposia sponsored by Fujirebio, Alzecure, and Biogen; and is a co‐founder of Brain Biomarker Solutions in Gothenburg AB (BBS), which is a part of the GU Ventures Incubator Program (outside submitted work). Kaj Blennow has served as a consultant or on advisory boards for Abcam, Axon, Biogen, Lilly, MagQu, Novartis, and Roche Diagnostics, and is a co‐founder of Brain Biomarker Solutions in Gothenburg AB (BBS), which is a part of the GU Ventures Incubator Program (outside submitted work). Jenna Najar, Sven Van der Lee, Erik Joas, Hanna Wetterberg, John Hardy, Rita Guerreiro, Jose Bras, Margda Waern, Silke Kern, Ingmar Skoog, and Anna Zettergren declare no competing interests.

## Supporting information

Supporting Information
