# Supplementary material for: Polygenic risk scores for Alzheimer's disease are related to dementia risk in APOE ɛ4 negatives
Source: Alzheimers Dement (Amst). 2021 Jan 22;13(1):e12142. doi: 10.1002/dad2.12142 (PMC7821873; doi:10.1002/dad2.12142)
Supplement: Supplementary file 1 — Supporting Information [file DAD2-13-e12142-s001.docx]

**Supplementary table 1.** Genetic variants included in the polygenic risk score and their individual effect on incident dementia.

| **Position (Chr:bp)** | **Genetic variants** | **Closest gene** | **Effect allele** | **Effect size^*^** | **RSQ**^†^ | **MAF** | **AD-PRS** | **HR (95% CI)**^‡^ | ***P* value** |
| --- | --- | --- | --- | --- | --- | --- | --- | --- | --- |
| 1:207802552 | rs4844610 | CR1 | A | 0.1466 | 0.993 | 0.198 | 39-SNPs AD-PRS | 1.16 (1.00-1.33) | .04 |
| 2:37515958 | rs876461 | PRKD3 | G | -0.0744 | 0.998 | 0.116 | 39-SNPs AD-PRS | 1.26 (1.03-1.54) | .02 |
| 2:127892810 | rs6733839 | BIN1 | C | -0.1693 | 1 | 0.386 | Both AD-PRSs | 0.98 (0.87-1.54) | .7 |
| 2:233981912 | rs10933431 | INPP5D | G | -0.1001 | 0.891 | 0.213 | Both AD-PRSs | 0.94 (0.82-1.09) | .4 |
| 2:127863224 | rs7584040 | BIN1 | T | 0.0862 | 0.996 | 0.208 | 1e^-5^ AD-PRS | 0.82 (0.71-0.93) | 2×10^-3^ |
| 2:186794162 | rs35695568 | FSIP2 | T | 0.1152 | 0.997 | 0.096 | 1e^-5^ AD-PRS | 1.09 (0.90-1.33) | .4 |
| 3:34413128 | rs76695866 | PDCD6IP | A | -0.182 | 0.965 | 0.042 | 1e^-5^ AD-PRS | 1.04 (0.77-1.40) | .8 |
| 3:44539183 | rs7617515 | ZNF852 | A | -0.0714 | 0.985 | 0.263 | 1e^-5^ AD-PRS | 1.05 (0.92-1.19) | .5 |
| 3:45101639 | rs6805148 | CLEC3B | A | 0.1293 | 0.993 | 0.066 | 1e^-5^ AD-PRS | 1.01 (0.80-1.27) | .9 |
| 3:86347434 | rs4574296 | CADM2 | A | 0.1013 | 0.993 | 0.078 | 1e^-5^ AD-PRS | 1.19 (0.97-1.46) | .1 |
| 4:110145870 | rs7686421 | COL25A1 | T | -0.1562 | 0.982 | 0.043 | 1e^-5^ AD-PRS | 1.31 (0.99-1.75) | .06 |
| 4:66245059 | rs28660482 | EPHA5 | A | 0.222 | 0.991 | 0.025 | 1e^-5^ AD-PRS | 0.99 (0,69-1.43) | .9 |
| 4:11027619 | rs4351014 | HS3ST1 | T | 0.0676 | 0.998 | 0.257 | 39-SNPs AD-PRS | 1.05 (0.92-1.19) | .5 |
| 5:139707439 | rs11168036 | HBEGF | T | 0.0754 | 0.976 | 0.485 | 1e^-5^ AD-PRS | 1.16 (1.04-1.30) | 8×10^-3^ |
| 5:29005985 | rs71618613 | SUCLG2P4 | A | 0.3901 | 0.852 | 0.013 | 1e^-5^ AD-PRS | 0.93 (0.54-1.59) | .8 |
| 6:108880646 | rs76185277 | FOXO3 | A | 0.2034 | 0.645 | 0.015 | 1e^-5^ AD-PRS | 1.36 (0.45-1.20) | .2 |
| 6:134389525 | rs9389138 | SLC2A12 | T | -0.0978 | 0.677 | 0.095 | 1e^-5^ AD-PRS | 0.99 (0.79-1.24) | .9 |
| 6:41034000 | rs114812713 | OARD1 | C | 0.298 | 0.831 | 0.016 | 1e^-5^ AD-PRS | 0.77 (0.48-1.21) | .3 |
| 6:47595155 | rs1385742 | CD2AP | A | 0.0876 | 0.971 | 0.354 | 1e^-5^ AD-PRS | 0.99 (0.88-1.12) | .9 |
| 6:32652196 | rs9275152 | HLA-DRB1 | T | 0.1319 | 0.955 | 0.147 | 39-SNPs AD-PRS | 1.08 (0.91-1.27) | .4 |
| 6:41129207 | rs143332484 | TREM2 | C | -0.495^§^ | 1 | 0.017 | 39-SNPs AD-PRS | 0.81 (0.53-1.25) | .3 |
| 6:41129252 | rs75932628 | TREM2 | C | -0.6989 | 1 | 0.001 | 39-SNPs AD-PRS | NA** | NA |
| 6:41154650 | rs9381040 | TREML2 | C | 0.0590 | 1 | 0.285 | 39-SNPs AD-PRS | 1.08 (0.95-1.22) | .3 |
| 6:47443806 | rs9381564 | CD2AP | A | -0.0830 | 0.992 | 0.274 | 39-SNPs AD-PRS | 1.03 (0.90-1.17) | .7 |
| 7:99971834 | rs1859788 | PILRA | A | -0.0652 | 0.980 | 0.310 | 39-SNPs AD-PRS | 0.88 (0.78-1.01) | .07 |
| 7:143103481 | rs56402156 | EPHA1 | G | 0.1024 | 0.964 | 0.206 | 39-SNPs AD-PRS | 1.01 (0.86-1.14) | .9 |
| 7:127426090 | rs117240937 | SND1 | A | -0.3122 | 0.848 | 0.009 | 1e^-5^ AD-PRS | 1.03 (0.52-2.06) | .9 |
| 7:143109139 | rs11767557 | EPHA1-AS1 | T | 0.1028 | 0.998 | 0.205 | 1e^-5^ AD-PRS | 1.00 (0.87-1.15) | .9 |
| 7:33721795 | rs143429938 | CLU/ MIR6843 | T | 0.3535 | 0.591 | 0.013 | 1e^-5^ AD-PRS | 0.56 (0.33-0.97) | .04 |
| 7:50322832 | rs9649710 | IKZF1 | A | -0.0676 | 0.990 | 0.387 | 1e^-5^ AD-PRS | 0.96 (0.86-1.08) | .5 |
| 7:6908860 | rs187857322 | OR7E136P | A | 0.4764 | 0.719 | 0.008 | 1e^-5^ AD-PRS | 0.87 (0.43-1.77) | .7 |
| 8:27468503 | rs867230 | CLU/ MIR6843 | A | 0.1333 | 0.982 | 0.417 | 1e^-5^ AD-PRS | 0.95 (0.85-1.07) | .4 |
| 8:98364076 | rs16895579 | LOC101927066 | A | 0.157 | 0.963 | 0.047 | 1e^-5^ AD-PRS | 1.07 (0.80-1.44) | .6 |
| 8:27219987 | rs73223431 | PTK2B | C | -0.0936 | 0.998 | 0.380 | Both AD-PRSs | 1.08 (0.96-1.22) | .2 |
| 8:27467686 | rs9331896 | CLU | C | -0.1269 | 0.987 | 0.421 | 39-SNPs AD-PRS | 0.95 (0.84-1.06) | .4 |
| 8:145154222 | rs34674752 | SHARPIN | G | -0.1068 | 0.789 | 0.039 | 39-SNPs AD-PRS | 1.29 (0.57-1.06) | .1 |
| 8:145158607 | rs34173062 | SHARPIN | G | -0.0896 | 0.647 | 0.084 | 39-SNPs AD-PRS | 1.06 (0.80-1.39) | .7 |
| 9:85450616 | rs6559689 | RASEF | T | 0.1585 | 0.827 | 0.047 | 1e-5 AD-PRS | 0.95 (0.73-1.25) | .7 |
| 10:11720308 | rs7920721 | ECHDC3 | A | -0.0782 | 0.986 | 0.367 | 39-SNPs AD-PRS | 1.06 (0.94-1.20) | .4 |
| 10:11721057 | rs12416487 | LOC105376412/ LOC105376413 | A | -0.085 | 0.984 | 0.345 | 1e^-5^ AD-PRS | 1.08 (0.95-1.22) | .2 |
| 10:66004362 | rs142366127 | DBF4P1 | T | 0.141 | 0.755 | 0.117 | 1e^-5^ AD-PRS | 1.09 (0.89-1.33) | .4 |
| 10:82271341 | rs1870148 | TSPAN14 | A | 0.0818 | 0.958 | 0.206 | 1e^-5^ AD-PRS | 1.06 (0.92-1.22) | .4 |
| 11:47380340 | rs3740688 | SPI1 | G | -0.0935 | 0.971 | 0.462 | Both AD-PRSs | 0.98 (0.87-1.10) | .7 |
| 11:60021948 | rs1582763 | MS4A2 | G | 0.1232 | 0.990 | 0.351 | Both AD-PRSs | 1.06 (0.94-1.20) | .3 |
| 11:85868640 | rs3851179 | PICALM | T | -0.1198 | 1 | 0.360 | Both AD-PRSs | 0.99 (0.88-1.12) | .9 |
| 11:121435587 | rs11218343 | SORL1 | T | 0.2053 | 0.979 | 0.047 | Both AD-PRSs | 0.99 (0.75-1.33) | .9 |
| 11:112559343 | rs72993825 | RPL23AP62 | T | -0.1763 | 0.841 | 0.060 | 1e^-5^ AD-PRS | 1.10 (0.85-1.44) | .5 |
| 11:131769402 | rs9787911 | NTM/ LOC107984413 | T | -0.0662 | 0.983 | 0.439 | 1e^-5^ AD-PRS | 0.89 (0.79-1.01) | .06 |
| 11:46804761 | rs11038990 | CKAP5 | T | 0.1232 | 0.864 | 0.101 | 1e^-5^ AD-PRS | 1.12 (0.90-1.39) | .3 |
| 11:47915299 | rs34467936 | NUP160 | A | 0.0905 | 0.984 | 0.360 | 1e^-5^ AD-PRS | 1.01 (0.90-1.14) | .9 |
| 11:76447123 | rs12291515 | GUCY2EP | A | -0.0986 | 0.965 | 0.114 | 1e^-5^ AD-PRS | 1.02 (0.85-1.23) | .8 |
| 12:127222883 | rs117394726 | LOC100996671 | A | -0.2193 | 0.643 | 0.037 | 1e^-5^ AD-PRS | 0.96 (0.69-1.33) | .8 |
| 13:113607147 | rs3011479 | MCF2L | A | 0.07 | 1 | 0.284 | 1e^-5^ AD-PRS | 0.98 (0.86-1.11) | .8 |
| 14:53391680 | rs17125924 | FERMT2 | A | -0.1222 | 0.992 | 0.099 | Both AD-PRSs | 0.95 (0.79-1.14) | .6 |
| 14:92936971 | rs11623019 | RIN3/SLC2A4 | T | 0.0913 | 0.993 | 0.191 | 39-SNPs AD-PRS | 0.99 (0.85-1.15) | .9 |
| 14:34446715 | rs1680666 | LOC107987210 | T | 0.0789 | 0.899 | 0.297 | 1e^-5^ AD-PRS | 0.95 (0.83-1.09) | .5 |
| 14:92938855 | rs12590654 | SLC24A4 | A | -0.0906 | 0.974 | 0.345 | 1e^-5^ AD-PRS | 0.99 (0.87-1.11) | .8 |
| 15:59045774 | rs593742 | ADAM10 | A | 0.0649 | 0.987 | 0.321 | 39-SNPs AD-PRS | 1.09 (0.96-1.23) | .2 |
| 15:63569902 | rs117618017 | APH1B | C | -0.0941 | 0.743 | 0.126 | 39-SNPs AD-PRS | 0.81 (0.67-0.98) | .03 |
| 15:59034174 | rs383902 | ADAM10 | T | -0.0698 | 1 | 0.351 | 1e^-5^ AD-PRS | 1.10 (0.98-1.24) | .1 |
| 16:19910313 | rs28588186 | GPRC5B | C | 0.088 | 0.983 | 0.171 | 1e^-5^ AD-PRS | 1.05 (0.90-1.23) | .5 |
| 16:70696626 | rs3752786 | MTSS2 | A | -0.0964 | 0.845 | 0.194 | 1e^-5^ AD-PRS | 1.00 (0.86-1.17) | .9 |
| 16:79355857 | rs62039712 | MAF | A | 0.1528 | 0.641 | 0.095 | 1e^-5^ AD-PRS | 1.03 (0.81-1.30) | .8 |
| 16:81779775 | rs34971488 | CMIP | A | 0.094 | 0.861 | 0.230 | 1e^-5^ AD-PRS | 1.04 (0,89-1.21) | .6 |
| 16:19808163 | rs7185636 | IQCK | T | 0.0786 | 0.993 | 0.144 | 39-SNPs AD-PRS | 0.99 (0.85-1.17) | .9 |
| 16:70694000 | rs4985556 | IL34 | C | -0.0867 | 0.899 | 0.122 | 39-SNPs AD-PRS | 0.98 (0.82-1.18) | .9 |
| 16:81773209 | rs12444183 | PLCG2 | A | -0.0507 | 1 | 0.350 | 39-SNPs AD-PRS | 1.00 (0.88-1.12) | .9 |
| 16:81900853 | rs3935877 | PLCG2 | T | 0.0623 | 1 | 0.073 | 39-SNPs AD-PRS | 1.13 (0.92-1.40) | .2 |
| 16:81942028 | rs72824905 | PLCG2 | C | 0.2701 | 1 | 0.008 | 39-SNPs AD-PRS | 1.02 (0.52-1.97) | .9 |
| 17:56409089 | rs2632516 | MIR142/ TSPOAP1-AS1 | C | -0.0748 | 0.988 | 0.448 | 1e^-5^ AD-PRS | 1.01 (0.90-1.13) | .9 |
| 17:61538148 | rs138190086 | CYB561 | A | 0.2535 | 0.950 | 0.017 | 1e^-5^ AD-PRS | 1.01 (0.66-1.52) | .9 |
| 17:73069108 | rs8064326 | KCTD2 | A | -0.1992 | 0.971 | 0.028 | 1e^-5^ AD-PRS | 0.57 (0.42-0.78) | 3×10^-4^ |
| 17:5138304 | rs75511804 | SCIMP | C | -0.0847 | 0.987 | 0.110 | 39-SNPs AD-PRS | 0.88 (0.73-1.05) | .1 |
| 17:44353222 | rs2732703 | KANSL1 | T | 0.0998^¶,#^ | 0.874 | 0.141 | 39-SNPs AD-PRS | 1.06 (0.89-1.27) | .5 |
| 17:47297297 | rs616338 | ABI3 | T | 0.362 | 1 | 0.010 | 39-SNPs AD-PRS | 0.93 (0.52-1.65) | .8 |
| 17:61560763 | rs4311 | ACE | T | -0.0481 | 1 | 0.462 | 39-SNPs AD-PRS | 1.05 (0.94-1.18) | .4 |
| 17:4805437 | rs72835061 | CHRNE | C | -0.0896 | 0.978 | 0.108 | 39-SNPs AD-PRS | 0.88 (0.77-1.01) | .07 |
| 19:1043638 | rs3752231 | ABCA7 | C | -0.1002 | 0.960 | 0.250 | 39-SNPs AD-PRS | 0.95 (0.85-1.07) | .4 |
| 19:51728477 | rs12459419 | CD33 | C | 0.0800 | 0.999 | 0.346 | 39-SNPs AD-PRS | 1.24 (0.97-1.59) | .09 |
| 19:1050874 | rs12151021 | ABCA7 | A | 0.1071 | 0.952 | 0.324 | 1e^-5^ AD-PRS | 1.05 (0.93-1.20) | .4 |
| 19:18558876 | rs8111708 | ELL | A | -0.0696 | 0.993 | 0.361 | 1e^-5^ AD-PRS | 0.93 (0.82-1.05) | .2 |
| 19:51727962 | rs3865444 | CD33 | A | -0.0804 | 1 | 0.346 | 1e^-5^ AD-PRS | 0.95 (0.85-1.07) | .4 |
| 20:54998544 | rs6014724 | CASS4 | A | 0.1319 | 0.881 | 0.063 | 1e^-5^ AD-PRS | 1.24 (0.97-1.58) | .09 |
| 20:54997568 | rs6024870 | CASS4 | G | 0.1279 | 0.901 | 0.062 | 39-SNPs AD-PRS | 1.05 (0.93-1.18) | .4 |
| 21:27473875 | rs2154481 | APP | C | -0.0505 | 0.988 | 0.458 | 39-SNPs AD-PRS | 0.85 (0.71-1.02) | .08 |
| 21:28148191 | rs2830489 | ADAMTS1 | T | -0.0837 | 0.946 | 0.285 | 1e^-5^ AD-PRS | 0.98 (0.86-1.11) | .7 |
| 21:37069610 | rs909441 | LOC100506403 | T | -0.1611 | 0.806 | 0.057 | 1e^-5^ AD-PRS | 0.93 (0.71-1.23) | .6 |
| 22:21926584 | rs138727474 | UBE2L3 | T | -0.2515 | 0.717 | 0.052 | 1e^-5^ AD-PRS | 1.03 (0.77-1.38) | .9 |

*Effect sizes from IGAP. ^†^RSQ (imputation quality) values of 1: genotyped SNP. ^‡^HR (95% CI) for the effect of the genetic variant in relation to dementia. ^§^Effect size from Sims et al. 2017. ^¶^Effect size for *APOE* ϵ4 carriers; Effect size for non-carriers=0.3145; ^#^Effect size from Jun et al. 2016. **Only two heterozygote carriers of risk allele in our sample.
